# Injury prevalence and safety habits of boda boda drivers in Moshi, Tanzania: A mixed methods study

**DOI:** 10.1371/journal.pone.0207570

**Published:** 2018-11-27

**Authors:** TuanDat Nguyen, João Ricardo Nickenig Vissoci, Treasure Joelson, Msafiri Pesambili, Michael Haglund, Charles J. Gerardo, Mark Mvungi, Catherine A. Staton

**Affiliations:** 1 Duke Global Health Institute, Duke University, Durham, North Carolina, United States of America; 2 Division of Emergency Medicine, Department of Surgery, Duke University School of Medicine, Durham, North Carolina, United States of America; 3 Division of Global Neurosurgery and Neurosciences, Department of Neurosurgery, Duke University Medical Center, Duke University, Durham, North Carolina, United States of America; 4 Kilimanjaro Christian Medical Center, Moshi, Tanzania; Technion Israel Institute of Technology, ISRAEL

## Abstract

**Background:**

Traffic crashes are a major cause of global morbidity and mortality disproportionately affecting low- and middle-income countries (LMICs). Motorcycle taxi (boda boda) drivers are particularly vulnerable because they are exposed to traffic risks with limited safety equipment. This study aims to characterize injury prevalence and safety habits among boda boda drivers, as well as ways to improve road traffic safety in LMICs.

**Methods:**

A cross-sectional mixed methods study was conducted with 300 boda boda drivers between 24 March and 3 April 2014 in urban Moshi, Tanzania. A convenience sample of participants was drawn from 25 of 58 registered boda boda stands and 2 of 31 unregistered stands. Data were analyzed using R, and content thematic analysis was performed and agreed upon by three investigators. Logistic regression models were used to evaluate the association between boda boda characteristics and injury risk.

**Results:**

In total, 300 drivers participated, of whom 148 (49.3%) had experienced a crash during their lifetime, and 114 (77.0%) sustained at least one injury. Only 27 of those injured (23.4%) were hospitalized. Of all participants, 220 (73.3%) reported consistent helmet usage, despite 285 participants (95.0%) agreeing that helmet usage reduces injury severity. From the 280 helmets observed, 231 (82.5%) were either damaged or fit improperly. Having a cracked helmet was associated with higher risk of being involved in a traffic crash. Owning a helmet with a proper fit was associated with reduced risk for a traffic crash (OR = 0.06) and road traffic injuries (OR = 0.07). A thematic analysis of boda boda drivers’ suggestions to increase road safety identified four intervention areas: 1) roadway infrastructure and traffic regulation, 2) road user attitudes and safe driving behaviors, 3) education and training, and 4) law enforcement.

**Conclusion:**

Our study demonstrates boda boda drivers’ safety behaviors and identifies four intervention areas that can be leveraged to increase overall road traffic safety. Unfortunately, while boda boda drivers are aware of ways to improve safety, adherence to safety habits remains low. Successful multi-sectoral interventions are needed to improve road safety for boda boda drivers in Tanzania.

## Introduction

Traffic crashes are a major cause of global morbidity and mortality, resulting in an estimated 20 million–50 million road traffic injuries (RTIs) and 1.2 million deaths per year [1]. Particularly in low- and middle-income countries (LMICs), RTIs place an enormous strain on monetary and human resources [2]. LMICs bear 85% of the global burden of motor vehicle fatalities and 90% of RTI-related disability adjusted life years (DALYs), despite accounting for only 32% of the world’s motor vehicle ownership in 2000 [3]. As LMICs become increasingly motorized, RTIs are projected to become the third highest cause of DALYs and the sixth leading cause of death by 2020 [4]. This is in part because in LMICs, there is a higher incidence of injury or death per crash; traffic safety laws and regulations are inconsistently enforced; and poor health infrastructure results in insufficient access to care [5–7]. As a result, LMICs are ill equipped to manage both the increased motorization and the resultant influx of RTIs.

Among the many types of road users, motorcycle riders are particularly vulnerable because they are exposed with limited protective equipment compared to the car and truck drivers with whom they share the road [8]. In a study on Tanzanian truck drivers’ opinion on road safety, drivers cited drunkenness, inattention, and sleepiness as reasons for reckless driving and crashes [9]. Despite ongoing efforts, research has shown that motorcycle riders face a higher risk of severe injury, morbidity, and mortality than other road users. For every kilometer travelled, motorcycle riders are 20 times more likely to die from a crash than other motor vehicle users [10]. Additionally, a recent study showed that 71% of all RTIs in rural Tanzania involved a motorcycle pre-intervention; however, incidence was not decreased after the intervention’s implementation [11]. The risks of riding a motorcycle are exacerbated by the fact that motorcycle riders tend to be more likely to engage in risky behaviors and make poor decisions on the road [12].

Despite the risks, many individuals in LMICs still choose motorcycles as their main mode of transportation for the benefits of convenience and low cost when compared to cost of owning an automobile. Motorcycle transportation became particularly prominent in countries affected by the Structural Adjustment Programs (SAPs) of the 1980s and 1990s. For example, structural adjustment in Nigeria increased motor vehicle prices by more than 200%, pricing car and minibus ownership out of reach for many and increasing the appeal of motorcycle transportation [13].

Additionally, due to poor roadway infrastructure in LMICs, motorcycles are often the “only means of transport to many streets, connecting roads, or villages” [14]. Even in areas with decent roadways, motorcycles are preferred for their effectiveness in bypassing traffic jams by weaving between stopped cars and passing slow vehicles [15]. This is especially pertinent given the rapid increase in the number of cars, leading to major traffic congestion [16]. The benefits of this quick and efficient means of transportation has led to the proliferation of motorcycle taxis in LMICs such as Tanzania, where they are referred to as ‘boda bodas’ [17]. Unfortunately, as boda bodas are increasingly used for commercial transportation, the incidence of motorcycle crashes is also increasing [18]. In the last decade alone, Tanzania experienced a fivefold increase in traffic-related fatalities [6]. As recently as 2016, an analysis of factors associated with road traffic collisions in Tanzania reported motorcycles to be the leading cause of traffic crashes, accounting for 53.4% of accidents in 6 public hospitals [19]. In fact, RTIs were involved in 43.9% of trauma cases and 66% of traumatic brain injury patients who presented at a hospital in Northwestern Tanzania, making RTIs the most common mechanism of injury [20, 21]. Data collected from police records of 300 motor vehicle collisions in Moshi, Tanzania, indicate that the involvement of vulnerable road users, such as motorcyclists, in a traffic crash was directly related to increased injury severity [22].

Though the reported incidence of RTIs is striking, it is widely accepted that the figures are underestimated, mostly due to limitations in data collection [23, 24]. For example, some hospital admissions figures do not account for the many motorcyclists who are fatally injured at the scene, nor do they adequately describe the fiscal and physical burden suffered by trauma victims who do not seek care [25–27]. Especially in developing countries such as Tanzania, data on the epidemiology of injury are only recently emerging, and the full extent of the burden of RTIs remains unknown [20].

The present study aims to define overall prevalence of non-fatal RTIs among boda boda drivers in Moshi, Tanzania, and to describe their safety practices, to further understand injury epidemiology in East Africa. This increased understanding of injury epidemiology can be leveraged to identify potential areas for further research and to develop effective intervention strategies to reduce the incidence of RTIs.

## Methods

### Study design

This was a cross-sectional mixed methods study of safety habits of boda boda drivers in Moshi, Tanzania, between 24 March and 3 April 2014. Ethical clearance was provided by the Kilimanjaro Christian Medical Center Ethics Committee as well as the Duke University Institutional Review Board. Our methods are described according to the GRAMMS guideline for mixed methods studies [28]. We conducted sequentially a quantitative portion quantifying safe habits and traffic crash indicators with a qualitative interview about suggestions for improvements to prevent traffic crashes.

### Quantitative approach—Survey about safe habits and road traffic experience

A survey of boda boda drivers’ safety habits and traffic crash experience was conducted between 24 March and 3 April 2014 in Moshi, Tanzania. We enrolled 300 boda boda drivers, chosen at convenience from 25 of 58 registered boda boda stands and 2 of 31 unregistered stands. Despite efforts to register boda boda stands, the specific population of boda boda drivers is unregistered and the total number of drivers (targeted population) is unknown. Thus, our sample size of 300 was adopted since it would be enough to study the prevalence of non-fatal traffic crashes for an unknown population, with 95% confidence and a 5% error margin, based on an average prevalence of 30%, as suggested in previous literature [29–31].

Stands were selected taking into account the spatial distribution that covered the Moshi Urban area, according to their locations. This approach was adopted to include stands that covered all the Moshi Urban geographical area, and also to cover areas for hotspots for motor vehicle crashes defined in a previous publication [22]. We included two unregistered stands because there were two areas of Moshi Urban that did not have a registered stand. It is important to highlight that although there is a differentiation between registered and unregistered stands, there is no specific difference in the regulations and registration of the boda boda drivers using each stand type.

A trained native Kiswahili-speaking research assistant approached boda boda drivers at boda boda stands at a random hour of the day and offered them participation in the research study. The research assistant invited all boda boda drivers present at the stand during a one-hour period of data collection at each stand. If they were interested in the research project, participants were provided informed consent and formally enrolled in the study. If they were not interested in participating, the research assistant invited the next driver until we reached the pre-specified sample size of 300. Each questionnaire took about 20 minutes. Participants received a 5,000 TSH (~2 USD) cell phone voucher as reimbursement for their time to participate in the research. The voucher is equivalent to the average value of one boda boda ride for about the length of the Moshi Urban research area. Trained data collectors administered the survey using a computerized, Internet-based survey tool (REDCAP) and tablet computers.

The questionnaire included questions about: (a) demographic information; (b) experience as a boda boda driver (e.g. number of years working as a boda boda driver); (c) injury history (e.g. have you ever suffered a traffic crash, have you ever suffered a RTI, have you had a near miss in the last month); and (d) safety habits (e.g. helmet use, helmet condition). Prior to data collection, we performed a pilot study with a sample of 5 boda boda drivers to ensure that the questions would be understood and that the survey/qualitative interviews could be done at their local stands during working hours. Some questions in the initial version of the survey were excluded after the pilot because they didn’t pertain to the Moshi setting. Additionally, piloting the survey allowed the research assistants to become familiar with the computer-based questionnaire administrate and navigate any Internet challenges. The full Swahili version of the questionnaire is included in the Appendix.

Data were analyzed using R Language for Statistical Computing [32]. Descriptive statistics were reported as frequencies, means with standard deviation ranges, and median with interquartile ranges, where appropriate. Three multivariate logistic regression models were built to evaluate the association of the safe habits of the boda boda drivers with: (1) having experience a traffic crash, (2) a RTI, and (3) a near miss in the last month. Predictors included helmet use behavior (helmet use and headlight use), helmet conditions (presence of cracks or a broken helmet chin strap, absence of face shield, obscured face shield, or improper fit of the helmet) and beliefs about helmet safety (helmet and helmet chin strap usage reduces risk of poor outcome and purchase of a new helmet after a crash event). Bivariate collinearity was evaluate by the polychoric correlation between predictors (see Appendix 2 for a correlation matrix). Multivariate collinearity was measured by the variance inflation factor (VIF). The variable presence of scratches was excluded as a predictor from the model multivariate models for indications of collinearity with presence of helmet cracks (high bivariate correlation and VIF above 3). Proper helmet fit showed a high correlation (R = 0.69) with helmet use behavior, but showed low VIF values (VIF = 1.2). Sensitivity analysis was conducted and models including proper helmet fit showed better fit (lower Akaike information criterion values—AIC) for the outcomes of having been involved in a traffic crash during their lifetime and a RTI. All other predictors showed low bivariate correlations and VIF values < 2. Proper helmet fit and belief that helmet usage reduces risk were excluded from the regression model of a near miss in the last month because they showed near zero variance. Regression models were controlled for age, hours of work per week, and years of experience as a boda boda driver.

### Qualitative approach—Suggestion for traffic crash improvement

Each boda boda driver was also interviewed with an open-ended question about their suggestions to improve safety and decrease traffic crashes. The interview occurred after the survey was administered and was initiated with the general question. Probing questions were used when needed to clarify the respondent’s suggestion for improvement. All interviews were conducted by the same research assistant in the local language, thus ensuring a standard procedure, and digitally recorded. Record files were transcribed using naturalized standard qualitative methods [33].

To analyze the qualitative data, we used a thematic content analysis. The qualitative interview transcripts were back translated, then independently reviewed and coded by two members (one from the United States and one from Tanzania) of the research team (TN and MP) using manual coding. Codes were grouped into categories, which were then reduced to themes through discussion and repeated review of interview scripts by the research team members (medical student, psychologist and physicians) [34]. Ambiguities and disagreements were resolved by discussion.

For each coded interview, we shared the resultant emerging themes with all the US and Tanzanian research team members for validation purposes. This validation helped confirm our findings and minimize the influences of personal bias. We continued discussing the emerging themes with the US and Tanzanian research teams for each coded interview until we reached a saturation point where no new themes were evident. Results for each category/theme are presented, along with their frequency of appearance in the participants’ responses.

## Results

### Demographic and clinical profile

Three hundred boda boda drivers operating in Moshi Urban, Tanzania, were surveyed during the study period. All drivers were male, with a mean age of 28 years. As seen in Table 1, boda boda drivers reported an average of 2.7 years of experience operating a motorcycle. Our sample of boda boda drivers also reported working for an estimated average of 85.23 hours per week.

**Table 1:** Characteristics of boda boda drivers.

Participants characteristics	Descriptive statistics
**Age, mean (SD)**	28.4 (7.1)
**Experience (years), mean (SD)**	2.7 (1.3)
**Hours worked per day, mean (SD)**	13.5 (2.0)
**Hours per week, mean (SD)**	85.2 (15.5)
**Suffered a RTC, N (%)**	148 (49.3)
**Suffered a RTI, N (%)**	114 (38.0)
**Near miss within 1 month, N (%)**	247 (82.3)
**RTI time of day, N (%)**	
Morning	26 (17.6)
Day	37 (25.0)
Evening	46 (31.1)
Night	36 (24.3)
**Number of injuries, N (%)**	
At least 1 injury	114 (38.0)
Multiple injuries	11 (3.6)
Injury location, N (%)*	
Head	37 (32.5)
Upper extremities	28 (24.6)
Lower extremities	42 (36.8)
Chest	5 (4.4)
Back	2 (1.8)
Patients hospitalized, N (%)*	27 (23.7)
Disability among injury patients, N (%)*	6 (5.3)
**Length of hospital stay (days), median (IQR)**	4 (2;10)
**Days of work missed, median (IQR)**	7 (2;30)

* % calculated for participants reporting at least one RTI (N = 114).

Of the 300 participants, 148 (49.3%) had been involved in a traffic crash. From the 148 traffic crashes, 118 (79.7%) occurred while the boda boda drivers were working. Crash rates increased as the day progressed, peaking in the evening hours. Of the 148 crashes, 26 (17.6%) occurred in the morning, 37 (25.0%) occurred during the day, 46 (31.1%) occurred in the evening, and 36 (24.3%) occurred at night, while 3 had missing information on the time of day. Near misses were highly prevalent, with 247 study participants (82.3%) reporting at least one near miss within the past month (Table 1).

The traffic crashes carried a high risk of injury, with 114 crash victims (38.0%) sustaining at least one injury and 11 crash victims sustaining multiple injuries. The most common injuries were injuries to the head and the lower and upper extremities (37.0%, 36.8%, and 24.6%, respectively) (Table 1). Despite the high incidence of injury, only 27 injured crash victims (23.7%) were hospitalized. As a result of the injuries, boda boda drivers missed an average of 7 (IQR 2–30) days of work.

### Safety habits

**Fig 1:**
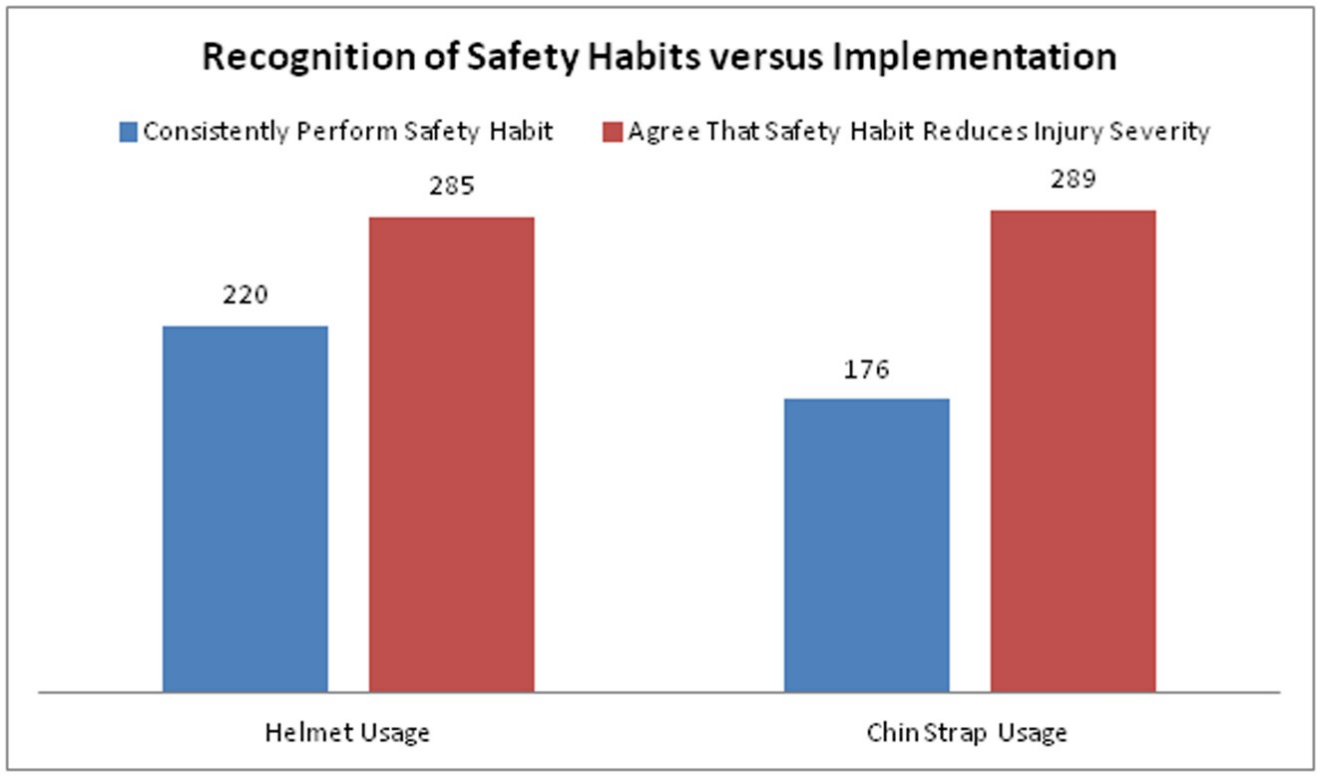
Knowledge versus usage of safety gear among motorcycle taxi drivers.

Only 220 participants (73.3%) reported consistent helmet usage despite 285 participants (95.0%) either agreeing or strongly agreeing that helmet usage is valuable in reducing injuries from a crash. Furthermore, only 176 participants (58.7%) reported consistent helmet strap usage despite 289 participants (96.3%) either agreeing or strongly agreeing that helmet strap usage helps reduce crash injuries (Fig 1).

**Fig 2:**
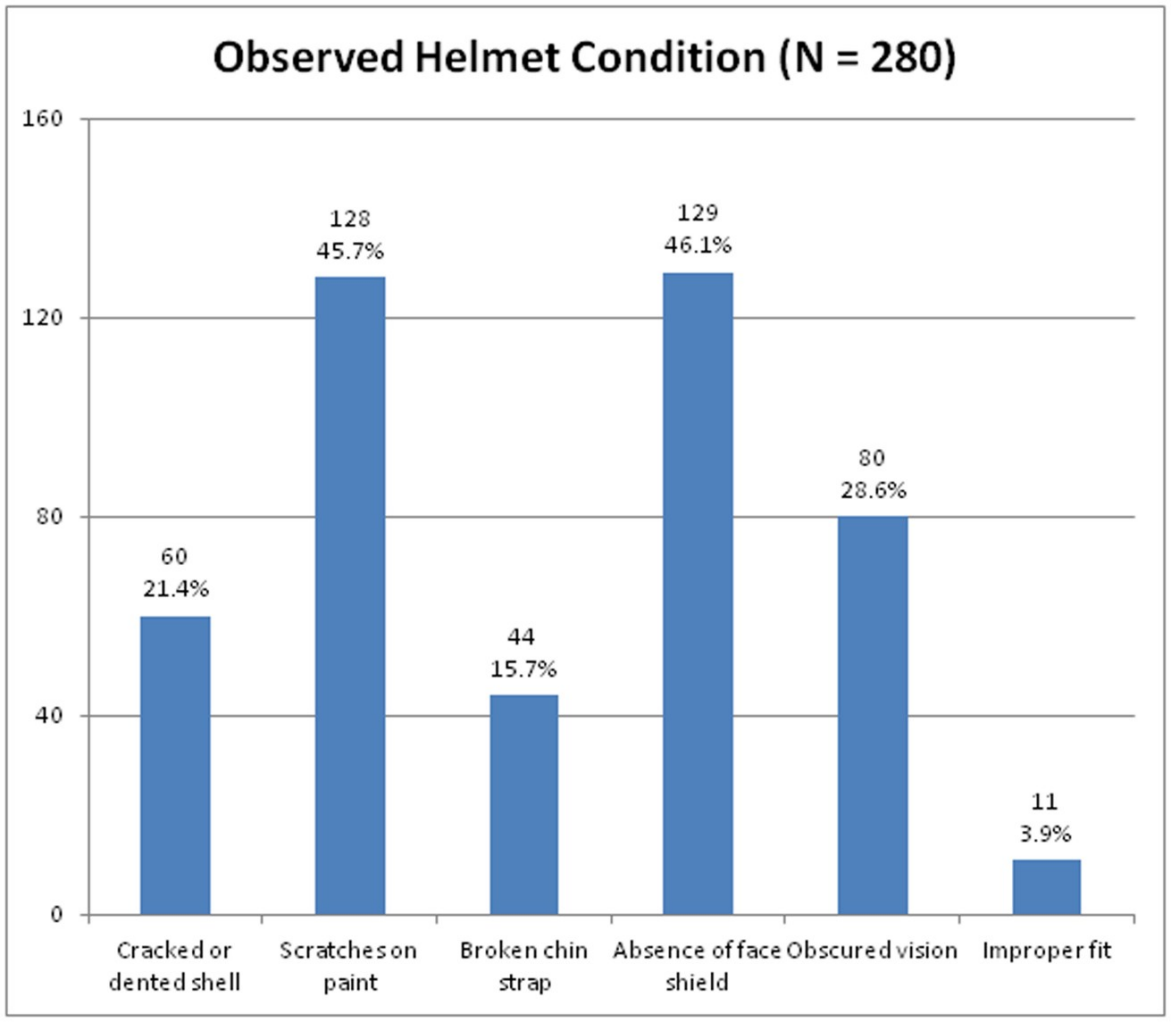
Observed helmet conditions among motorcycle taxi drivers.

Of the 280 helmets observed, 60 helmets (21.4%) had cracked or dented shells, 128 helmets (45.7%) had scratches in the paint, and 44 helmets (15.7%) had broken chin straps. Only 151 helmets (53.9%) featured face shields. Scratches, paint, and graphics obscured driver vision on 53.0% (n = 80) of those face shields (Fig 2).

In terms of boda boda conspicuity, only 109 drivers (36.3%) drove with their headlights on at all times. However, consistent headlight usage increased to 91.0% (n = 273) after dark. When asked to describe their colleagues’ driving habits, a staggering 97.3% (n = 292) believed that their colleagues were engaged in risky driving behaviors at least occasionally. Only 2.7% (n = 8) of participants believed that their colleagues never engaged in risky driving.

Boda boda drivers’ responses showed that having a cracked helmet was associated with a 2 times higher risk of having been involved in a traffic crash, while drivers who owned a helmet with a proper fit were less likely to have been in a traffic crash (OR = 0.06). Regarding injury, having a helmet with a proper fit was also associated with a lower risk of RTIs (OR = 0.07). As for near misses, participants who reported using a chin strap buckle when using a helmet were less likely to report a near miss in the last month. Interestingly, participants who used headlights when driving at night showed a higher risk of near misses (Table 2).

**Table 2:** Association between boda boda safety habits and road traffic outcomes.

	Traffic crash			Road traffic injury			Near miss		
Variables	Yes	No	OR (CI 95%)	Yes	No	OR (CI 95%)	Yes	No	OR (CI 95%)
**Age, mean (SD)**	27.2 (6.31)	29.5 (7.68)	0.95 (0.91;0.99)*	27.0 (6.6)	29.6 (7.3)	0.95 (0.91;0.99)*	28.4 (6.9)	30.5 (8.3)	0.97 (0.92;1.03)
**Experience (years), mean (SD)**	2.7 (1.2)	2.8 (1.4)	1.01 (0.81;1.25)	2.7 (1.2)	2.8 (1.4)	1.03 (0.81;1.30)	2.8 (1.3)	2.6 (1.3)	1.19 (0.89;1.65)
**Hours per week, mean (SD)**	13.3 (2.2)	12.8 (1.8)	1.02 (0.99;1.04)	13.7 (2.3)	12.8 (1.8)	1.02 (0.99;1.04)	13.3 (2.1)	12.3 (1.7)	1.03 (1.00;1.05)*
**Use helmets, N (%)**	85 (75.9)	113 (84.3)*	1.04 (0.49;2.23)	62 (74.7)	136 (83.4)	1.04 (0.48;2.33)	168 (79.6)	30 (85.7)	1.43 (0.41;4.38)
**Chin strap use, N (%)**	95 (84.8)	111 (82.8)	0.91 (0.51;1.64)	72 (86.7)	134 (82.2)	1.18 (0.64;2.22)	171 (81.0)	35 (100)	0.23 (0.06;0.64)**
**Obscured vision, N (%)**	73 (65.2)	95 (70.9)	0.68 (0.37;1.26)	54 (65.1)	114 (69.9)	0.83 (0.38;1.40)	148 (70.1)	20 (57.1)	1.28 (0.54;2.97)
**Absence of face shield, N (%)**	65 (58.0)	84 (62.7)	0.85 (0.48;1.50)	47 (56.6)	102 (62.6)	0.80 (0.44;1.47)	124 (58.8)	25 (71.4)	0.71 (0.29;1.67)
**Proper fit, N (%)**	103 (92.0)	133 (99.3)*	0.08 (0.00;0.52)*	75 (90.4)	161 (98.8)*	0.09 (0.01;0.48)**	201 (95.3)	35 (100)	-
**Headlight use during the day, N (%)**	41 (36.6)	50 (37.3)	1.08 (0.62;1.89)	32 (38.5)	59 (36.2)	1.23 (0.68;2.21)	76 (36.0)	15 (42.9)	0.76 (0.34;1.68)
**Headlight use during the night, N (%)**	103 (92.0)	119 (88.8)	1.75 (0.70;4.64)	78 (93.9)	144 (88.3)	2.55 (0.91;8.58)	193 (91.5)	29 (82.9)	3.79 (1.18;11.51)*
**Helmet use belief, N (%)**	109 (97.3)	137 (94.7)	2.60 (0.62;14.72)	81 (97.6)	155 (95.1)	2.93 (0.61;23.05)	201 (95.3)	35 (100)	-
**Strap use belief, N (%)**	110 (98.2)	130 (97.0)	3.16 (0.44;37.3)	2 (97.6)	4 (97.5)	2.01 (0.27;22.48)	206 (97.6)	34 (97.1)	1.81 (0.08;16.94)

* P-value <0.05;

** P-value <0.01.

### Suggested improvements

When asked how road traffic safety for boda bodas could be improved, the most common suggestions were stricter enforcement of helmet usage (81%, n = 243), improved driver training for boda boda drivers (79.7%, n = 239), usage of reflective vests for drivers (79.7%, n = 239), increased awareness of boda bodas by other vehicles (68%, n = 204), and improved road conditions (58.3%, n = 175). Other suggestions included increased numbers of pedestrian walkways (54%, n = 162), improved traffic regulation (54%, n = 162), better road lighting (45.3%, n = 136), lower traffic volume (44.3%, n = 133), and the creation of a boda boda traffic lane (43%, n = 129). These suggested improvements can be grouped into four main themes: improved roadway infrastructure and traffic regulation, road user attitudes and safe driving behaviors, increased education and training, and stricter law enforcement (Table 3).

**Table 3:** Thematic analysis of boda boda driver short answer responses.

Theme	Specific responses	Surveys citing category N (%)	Surveys citing theme N (%)
**Improved roadway infrastructure and traffic regulation**	Improved road conditions	178 (59.3)	911 (41.1)
	More pedestrian walkways	162 (54.0)	
	Improved traffic regulation	162 (54.0)	
	Increased road lighting	137 (45.7)	
	Lower traffic volume	133 (44.3)	
	Creation of boda boda traffic lane	129 (43.00	
	Wider roads	6 (2.0)	
	Road signs	4 (1.3)	
**Road user attitudes and safe driving behaviors**	Helmet usage	243 (81.0)	948 (42.8)
	Reflective vests for boda boda drivers	240 (80.0)	
	Awareness of boda bodas	205 (68.3)	
	Carefulness	71 (23.7)	
	Reduce alcohol consumption	55 (18.3)	
	Observation of road rules and regulations	53 (17.7)	
	Reduce driving speed	53 (17.7)	
	Respect for other road users	20 (6.7)	
	Confident driving	4 (1.3)	
	Reduce distracted driving (negligence, having other thoughts)	4 (1.3)	
**Increased education and training**	Improved boda boda driver training	242 (80.7)	344 (15.5)
	General education for road users	102 (34.0)	
**Stricter law enforcement**	Inspect drivers and verify licenses	5 (1.7)	12 (0.5)
	Reduce police corruption	4 (1.3)	
	Punishment/fines	3 (1.0)	

### Improved roadway infrastructure and traffic regulation

These suggestions center on the idea of creating a driving environment conducive to road safety, and point to the fact that Tanzania’s current roadway infrastructure is unsatisfactory to that end. Roads may be considered low quality due to either low design standards, poor workmanship, or both. Participants made such suggestions as increasing road lighting, which would improve boda boda visibility in low-light conditions, creating a designated boda boda traffic lane, which would reduce the likelihood of collisions with other road users, and creating more lanes to help decrease traffic volume. We define traffic volume as the count of vehicles passing by the stand during the data collection time. Responses that fell into this theme included, “Traffic lights should be installed on the roads,” “Roads should be better constructed because they have low quality,” “Roads are so narrow, so they should widen the roads,” and “Road signs should be visible.”

### Road user attitudes and safe driving behaviors

These suggestions center on the idea of encouraging boda boda drivers to take responsibility for their own safety by adopting safer driving habits while avoiding risky driving habits. Suggestions included more consistent usage of helmets and reflective vests, careful and respectful driving, and the reduction of overall alcohol consumption in the population, all of which are factors of road safety within the driver’s control. Responses that were assigned to this category included, “Boda boda drivers should wear helmets all the time when they are on the road,” “Boda boda drivers need to put on the headlights during the day so they can be visible,” “Drivers should reduce speed and alcohol use during work hours,” and “Drivers should wear reflectors.”

### Increased education and training

These suggestions center on the idea of educating road users how to drive safely and reduce their risk for injuries. Suggestions include safety seminars, improved boda boda training programs, and general education for all road users. Responses assigned to this category included, “Seminars to educate all kinds of drivers,” “Education should be provided [so road users would] drive in average speed,” “Frequent education to all drivers,” and “Education should be provided [to] all road users.”

### Stricter law enforcement

These suggestions center on the idea that stricter enforcement of existing traffic laws will increase adherence to those laws. The suggestions called for the inspection and verification of driving licenses, increased punishments and fines for violating traffic laws, and controlling police corruption. Responses that fit this theme included, “Police should fulfill their duties and stop being corrupt,” “Police should do their responsibility,” “Police should make sure that all drivers have a driving license,” and “[Boda boda] Drivers should be inspected for their driving license.”

## Discussion

Road traffic injuries cost LMICs over $100 billion annually in both direct medical costs and lost wages [1]. Additionally, RTIs are the second leading cause of death among men between 15 and 49 years old, an economically productive age group, which reduces the national labor force and productive capacity [34]. The present study adds to the growing literature on road traffic safety by characterizing injury prevalence and safety habits of boda boda drivers in Moshi, Tanzania. Furthermore, the study identifies four major intervention points that can be leveraged in order to improve road traffic safety.

Due to the nature of their work, boda boda drivers are prone to high levels of fatigue and stress. A previous study found that boda boda drivers work incredibly long hours, which can result in the accumulation of physical fatigue; additionally sleepiness and inattention were named common crash causes by Tanzanian truck drivers [9]. The study also shows a high frequency of boda boda crashes and near misses; this constant exposure to risky situations can cause mental fatigue and stress [35]. Previous studies have shown that fatigue can impair a driver’s ability to control both speed and steering [36]. Furthermore, fatigue can slow reaction time and reduce a driver’s ability to maintain proper lane position [37]. Thus, prolonged periods of boda boda operation may increase the RTI risk by impairing safe driving ability. Unfortunately, Tanzanian boda boda drivers have a monetary incentive to work as many hours as possible, which increases the likelihood that boda boda drivers will continue to operate their vehicles despite fatigue and stress.

Not only are boda boda drivers incentivized to work longer hours, but they are also incentivized to engage in riskier driving behaviors, such as speeding and weaving through traffic, which reduces gas consumption and allows boda boda drivers to serve more customers [38]. Speeding has been strongly associated with higher severity of non-fatal injuries in urban settings [39].

In addition to the inherently risky nature of boda boda operation, the data show that the average boda boda driver has had less than three years of experience. Previous research has shown that inexperienced drivers are overconfident in their driving ability [40] and are slower to recognize and respond to potential hazards [41]. Conversely, experienced drivers tend to approach hazards at appropriate speeds and are less likely to crash [42, 43]. The high number of inexperienced boda boda drivers on the road may reduce overall road traffic safety and contribute to higher rates of road traffic crashes and RTIs.

Notably, most of the reported crashes occurred in the evening hours, when the glare of the setting sun may have impaired both the vision and visibility of the boda boda drivers. Previous research has shown that “periods of extreme sun glare reduce the ability to safely operate a motor vehicle,” particularly at intersections [44]. Similarly, boda boda visors, when present, were often damaged, further limiting the vision of boda boda drivers.

Another issue regarding boda boda visibility is the usage of conspicuity measures. One study found that usage of conspicuity measures such as reflective clothing, continuous headlight usage, and white helmets reduced the risk of crash by 37%, 27%, and 24%, respectively [45]. In light of this data, it is heartening to report that the vast majority of boda boda drivers in this study recognized the importance of wearing helmets and reflective vests, with 81% and 80% of respondents citing these as recommendations to improve road safety, respectively. However, further research is required to find effective ways of translating knowledge to actual practice, as evidenced by a study by Sumner et al. [24], which found that even free distribution of safety equipment increased usage only modestly, even after participants received a brief education session to explain the safety benefits of safety equipment. The study population of this and the Sumner study are similar, boda boda drivers from registered stands in the Moshi Urban region, so these high rates of knowledge about conspicuity measures could be retained knowledge from the Sumner educational intervention.

In the event of a crash, boda boda drivers often suffer both physical and financial hardships. Our data show that the vast majority of injured crash victims do not receive the care they need; in the present study, only 23.7% of injured crash victims were hospitalized, and only 13.2% received any rehabilitation. While we were not able to compile the cost of treatment for our boda boda drivers, a tertiary hospital study in Uganda estimated the cost of medical care per injured patient to be roughly 300 USD, or the monthly salary of a registered nurse. This estimation excludes other expenses such as travel, food, medication, and loss of revenue [15]. Crash victims were also unable to work for an average of 27.5 days. For crash victims who received treatment, this could mean financially crippling hospital and rehabilitation bills with no income to pay for the treatment. Those who did not receive treatment still faced the bleak reality of nearly a month without income.

Surprisingly, despite the general consensus among boda boda drivers that helmets and helmet straps reduce the risk of injury in the event of a crash, only 73.3% of drivers reported wearing a helmet consistently and only 58.7% consistently wore chin straps. Regarding the quality of the helmets, many of the helmets were found to have cracks, dents, scratches, and broken chin straps. It is reasonable to assume that damaged helmets are unreliable and offer less protection in the event of a crash. Unfortunately, the data show that boda boda drivers are unlikely to replace their helmets when they are damaged. This is likely due to the cost of buying a new helmet, which is more than twice the amount boda boda drivers would ideally spend. Some helmets not only offer less protection, but may even increase the risk of a crash, as is the case with the many helmets featuring face shields that obscure driver vision with scratches, paint, or graphics.

The survey data suggest four main areas of focus to increase road traffic safety, which reflect the commonly cited three E’s of road safety: engineering, education, and enforcement [46]. The first is improved roadway infrastructure and traffic regulation, which deals with creating a safer driving environment for boda bodas through the creation of a boda boda–specific traffic lane, increased road lighting, and improved road quality. Our conclusions are corroborated by previous studies, Polus et al [47] demonstrated that high-quality infrastructure can reduce crash rates by 44% while Zegeer and colleagues [48] found that shoulder widening reduced crash rates by 49%. However, other studies have shown that improved road conditions may lead to more speeding and therefore more crashes, as compared to a control group [11]. Therefore, caution must be taken when implementing new interventions to prevent unintended negative consequences. The second area is addressing road user attitudes and safe driving behaviors by promoting respectful, careful, and confident driving while calling for a reduction in both alcohol usage and driving speed. Previous studies found similar alcohol and speed risk factors; in the United States, alcohol is implicated in 41% of fatal injuries from traffic crashes, and the National Highway Traffic Safety Administration found speeding was involved in 30% of traffic-related fatalities [49, 50]. The third aspect is increased education and training, which seeks to offer road users the skills necessary to reduce their risk for RTIs. A pre-post experimental study conducted at Clemson University found that even a short training video can improve drivers’ safety skills and reduce traffic accidents due to run-off-road events, further verifying our results [51]. The fourth focus is stricter law enforcement to ensure that road users adhere to traffic safety laws. For instance, Vaa [52] demonstrated that increased police enforcement reduced average motor vehicle speeds by 0.9–4.8 km/h. Similarly, increased penalties or fines are effective in combating traffic violations [53]. Drivers may be motivated to adhere to the law to avoid financial loss. Areas of increased police enforcement enjoyed a reduction in vehicle speed and proportion of speeding drivers for several weeks after the period of increased reinforcement, with some areas experiencing a “time-halo” effect of up to 8 weeks. Clearly, stricter law enforcement can help reduce risky driving behaviors. Interventions should be designed to address all four of these focus areas in order to efficiently improve road traffic safety as multifaceted road traffic interventions have been shown to be more effective [54].

## Limitations

Some limitations to this study should be acknowledged when evaluating our results. First, as this was a survey of active boda boda drivers, it is subject to a significant survivor bias. As such, we did not focus on injuries, since those with fatal injuries and severe life and job-threatening injuries would have been missed, but on safety and safety behaviors since our population was more likely to represent the best safety profile among the survivors. This survivor bias also limits our ability to make accurate disability, missed work, and lost wage estimates given more severe injuries were excluded; still, with our safer, surviving population, these estimates are conservative estimates. Secondly, our method, using self-report and personal opinion about road safety improvements, has intrinsic bias in self-reporting and understanding of road safety. Even in light of this, we found significant differences between knowledge and practice of safety behaviors highlighting that knowledge alone is insufficient for safe behavior. Similarly, many boda boda drivers cited external causes for crashes most likely due to a perspective bias, yet some participants did cite that alcohol use among boda boda drivers was a significant road safety concern. And while participants cite improvements like ‘need for road quality improvements,’ these suggestions should be taken with caution; while improved lighting has obvious safety impact, improved road quality has variable impact as it can be associated with increased speeds therefore more injuries [44].

## Conclusion

The present study demonstrates the high prevalence of traffic crashes and injuries that make boda boda driving an inherently risky occupation. Fatigue, stress, and inexperience can increase the risk of injury for boda boda drivers. Traffic crashes and injuries can cause both physical and financial hardship for the crash victim. Many intervention points can be leveraged to reduce the risk of injury and increase overall road traffic safety. Unfortunately, while boda boda drivers are aware of ways to improve safety, adherence to safety habits remains low. Successful interventions will bridge the gap between knowledge and practice of safety habits.

## Supporting information

S1 Appendix Boda boda survey codebook. (PDF)

S2 Appendix Correlation matrix of predictors of road traffic outcomes. (CSV)
