# Supplementary material for: Injury prevalence and safety habits of boda boda drivers in Moshi, Tanzania: A mixed methods study
Source: PLoS One. 2018 Nov 27;13(11):e0207570. doi: 10.1371/journal.pone.0207570 (PMC6258469; doi:10.1371/journal.pone.0207570)
Supplement: S1 Appendix — (PDF) [file pone.0207570.s001.pdf]

Duke University
Duke Office of Clinical Research

Tanzania Survey

Codebook

Data Dictionary Codebook

10/22/2018 3:31pm

Collapse all instruments

#	Variable / Field Name	Field Label<br>*Field Note*	Field Attributes (Field Type, Validation, Choices, Calculations, etc.)
Instrument: **Pilot** (pilot)			Collapse
1	participant_id	Participant ID	text
2	pilot_id	Nambari ya mshiriki	text, Identifier
3	complete	Ni nani aliye kamilisha utafiti huu/Dodoso hii?	radio, Identifier<br>0 0<br>1 1<br>2 2<br>3 3<br>4 4
4	circulator	Je, mahojiano haya ya Bodaboda yamefanyika kutoka eneo la maegesho au mzunguko	radio<br>0 Maegesho ya bodaboda<br>1 Bodaboda kwenye mzunguko
5	parkingspot	Tafadhali andika jina la eneo la maegesho ya bodaboda ambapo mahojiano haya yamefanyika	text, Identifier
6	interview_location	D0. Kabla ya kuanza mahojiano ratibu coordinates za eneo lako la sasa na nakala yako iandike katika sehemu hii. Hakikisha usahihi katika upande wa kulia juu ya Windo na endelea kubonyeza kifungo katika ukurasa ili ijiReload mpaka kufikia usahihi uwe chini ya mita 50.<br>*CLICK HERE TO GET COORDINATES:*	text
7	accurate	D1. Je, coordinates ni sahihi kwa kiasi gani?	text

8	consent	Section Header: *Demographics*<br>D2. MSOMEE HII DEREVA WA BODABODA- Ahsante kwa kukubali kuzungumza na mimi leo . Utafiti huu unafanywa na timu ya watafiti kutoka katika hospitali ya KCMC ya Mjini moshi Tanzania . Lengo la utafiti huu ni kutaka kutambua maeneo hatari zaidi ndani ya Moshi ambayo ajali nyingi za barabarani hutokea mara kwa mara.Maoni yako yatasaidia kuyafanya maeneo haya kuwa salama zaidi. Umechaguliwa kushiriki katika utafiti huu kwa sababu unatumia barabara mara kwa mara.Hakuna utambulisho wako wowote ambao utachukuliwa na maoni yako yataendelea kuwa siri (bila kuwa na majina yako). Utafiti huu utachukua muda wa dakika 10 hadi 15 ya muda wako na utafidiwa muda wako kwa kiasi cha vocha ya TSH 5000.Je, Ungependa kushiriki katika utafiti huu?	yesno<br>1 Yes<br>0 No<br><br>Custom alignment: RH
9	not_consent<br>Show the field ONLY if:<br>[consent]='0'	D2a . Kwa nini umekataa kushiriki katika utafiti huu?	text
10	age<br>Show the field ONLY if:<br>[consent]='1'	D3.Una umri gani?	text (number, Min: 18, Max: 100)
11	male<br>Show the field ONLY if:<br>[consent]='1'	D4 . jinsia	radio<br>0 Ke<br>1 Me<br>99 Haifahamiki<br><br>Custom alignment: RH
12	occupation<br>Show the field ONLY if:<br>[consent]='1'	D5 . Kazi yako kuu ni ipi? (Ni aina ipi ya kazi ambayo inakupatia kipato kukubwa zaidi ?)<br>*Choose 'Transportation' if they say that working as a boda-boda driver is their main occupation.*	radio<br>0 Elimu<br>1 Kilimo<br>2 Biashara<br>3 Huduma za viwandani<br>4 Afya<br>5 Ujenzi<br>6 Mkahawa/baa<br>7 Usafirishaji

13	thanks<br>Show the field ONLY if:<br>[occupation]="0" or [occupation]="1" or [occupation]="2" or [occupation]="3" or [occupation]="4" or [occupation]="5" or [occupation]="6"	Ahsante kwa kuzungumza na mimi, lakini sisi tunahitaji kuzungumza na wale wanaofanya kazi katika sekta ya usafirishaji tu . Ahsante!	descriptive
14	hours_wkvehicle<br>Show the field ONLY if:<br>[consent]='1'	D6 . Ni masaa mangapi kwa siku unayotumia kufanya kazi na BodaBoda hii?	text (number, Min: 1, Max: 23)
15	day_wkvehicle<br>Show the field ONLY if:<br>[consent]='1'	D7 .Ni Siku ngapi katika wiki unazotumia kufanya kazi na bodaboda hii ?	text (number, Min: 1, Max: 7)
16	time_start_wk<br>Show the field ONLY if:<br>[consent]='1'	D8 . Kwa kawaida kila siku unaanza shughuli zako za usafirishaji saa ngapi ?<br>*HH:MM*	text (time, Min: 00:00, Max: 23:59)
17	time_stop_wk<br>Show the field ONLY if:<br>[consent]='1'	D9 .Kwa kawaida kila siku huwa unamaliza shughuli zako za usafirishaji saa ngapi?<br>*HH:MM*	text (time, Min: 00:00, Max: 23:59)
18	time_boda_bodadr<br>Show the field ONLY if:<br>[consent]='1'	D10 .Ni miaka mingapi sasa toka uanze shughuli ya bodaboda ?<br>*if they say less then 1 year you should just type 1*	text (number, Min: 1, Max: 90)
19	vehicle_home<br>Show the field ONLY if:<br>[consent]='1'	D11 . Ni aina ipi kuu ya usafiri ambayo unatumia wakati unapokuwa hauko kazini?	dropdown<br>0 Wa umma<br>1 Pikipiki ya magurudumu-3(Bajaji)<br>2 Usafiri binafsi<br>3 Usafiri wa mtu mwingine<br>4 Bodaboda yako binafsi<br>5 Bodaboda ya mtu mwingine<br>6 Baiskeli<br>7 Kwa miguu<br>8 Kwa mnyama<br>9 Dala-dala<br>90 Nyingine(fafanua)
20	vehicle_home_other<br>Show the field ONLY if:<br>[vehicle_home]= '90'	D11a . Ni aina zipi nyingine za usafiri unazotumia wakati unapokuwa hauko kazini?	text

21	hours_hmvehicle<br>Show the field ONLY if:<br>[consent]='1'	D12 .Ni masaa mangapi kwa siku ambayo unatumia usafiri huo?	text (number, Min: 1, Max: 23)
22	wide_travel<br>Show the field ONLY if:<br>[consent]='1'	D13 . Tunataka kuelewa ni jinsi gani kwa kawaida huwa usafiri ukiwa mjini Moshii. Ni mahali gani huwa unakwenda mara nyingi zaidi ukitokea katika maegesho haya ya bodaboda?<br>*Make sure to type in the reference point, village, and sector of the location they identify.*	text
23	crash_lifetime<br>Show the field ONLY if:<br>[consent]='1'	Section Header: *Crash Information*<br>C1. Katika maisha yako , umewahi kuhusika katika ajali ya barabarani ?	radio<br>0 Hapana<br>1 Ndiyo<br>99 Hifahamiki<br>Custom alignment: RH
24	rem_date_crash<br>Show the field ONLY if:<br>[crash_lifetime]='1'	C2. Je, unakumbuka tarehe ya ajali?	yesno<br>1 Yes<br>0 No<br>Custom alignment: RH
25	date_crash<br>Show the field ONLY if:<br>[rem_date_crash]="1"	C2a. Ilikuwa tarehe ngapi?<br>*ddmmyy*	text (date_dmy, Min: 1960-01-01, Max: 2014-02-01), Identifier
26	day_crash<br>Show the field ONLY if:<br>[rem_date_crash]="0"	C2b. Ni Siku gani ya wiki ambapo ajali ilitokea?	dropdown<br>0 Jumatatu<br>1 Jumanne<br>2 Jumatano<br>3 Alhamisi<br>4 Ijumaa<br>5 Jumamosi<br>6 Jumapili<br>7 Haifahamiki
27	time_crash<br>Show the field ONLY if:<br>[crash_lifetime]='1'	C3. Ni wakati gani ajali ilitokea	dropdown<br>0 Asubuhi<br>1 Mchana<br>2 Jioni<br>3 Usiku<br>99 Haifahamiki

28	type_crash<br><br>Show the field ONLY if:<br>[crash_lifetime]='1'	C4. Ni kwa namna gani ulihusika katika ajali hiyo	dropdown<br>0 Mtembea kwa miguu<br>1 Mpanda baisikeli<br>2 Dereva wa pikipiki ya magurudumu-3(bajaji)<br>3 Abiria wa pikipiki ya magurudumu-3(Bajaji)<br>4 Dereva wa bodaboda<br>5 Abiria wa bodaboda<br>6 Dereva wa gari dogo<br>7 Abiria wa gari dogo<br>8 Dereva wa basi<br>9 Abiria wa basi<br>10 Dereva wa lori<br>11 Abiria wa lori<br>12 Dala-Dala Driver<br>13 Dala-Dala Passenger<br>90 Yingine(fafanua)<br>99 Hifahamiki
29	type_crash_other<br><br>Show the field ONLY if:<br>[type_crash]= "90"	C4a. Ni aina ipi nyingine ya ajali ya barabarani ambayo umewahi kuhusika?	text
30	work_crash<br><br>Show the field ONLY if:<br>[crash_lifetime]='1'	C5. Je ulikuwa kazini kama dereva wa boda boda wakati ulipopata ajali hiyo?	radio<br>0 Hapana<br>1 Ndiyo<br>99 Haifahamiki<br><br>Custom alignment: RH
31	injured_crash<br><br>Show the field ONLY if:<br>[crash_lifetime]='1'	C6. Je, katika ajali hiyo uliumia/ulipata majeraha?	radio<br>0 Hapana<br>1 Ndiyo<br>99 Haifahamiki<br><br>Custom alignment: RH

32	injuries_crash<br>Show the field ONLY if:<br>[injured_crash]='1'	C7. Ni aina gani ya majeraha ambayo uliumia/uliyopata?	checkbox<br>0 | injuries_crash___0 | Majeraha ya kichwani<br>1 | injuries_crash___1 | Majeraha ya usoni<br>2 | injuries_crash___2 | majeraha ya uti wa mgongo<br>3 | injuries_crash___3 | Majeraha ya juu juu ya kifuani<br>4 | injuries_crash___4 | Majeraha yaliyochimbika kwenye kifua<br>5 | injuries_crash___5 | Vidonda vya juu juu katika fumbatio/tumbo[illegible]<br>6 | injuries_crash___6 | Majeraha yaliyochimbika katika fumbatio/tumbo[illegible]<br>7 | injuries_crash___7 | Kuvunjika mikono<br>8 | injuries_crash___8 | Kuvunjika miguu<br>9 | injuries_crash___9 | Kukatika mikono<br>10 | injuries_crash___10 | Kukatika miguu<br>90 | injuries_crash___90 | Nyingine(fafanu[illegible]<br>99 | injuries_crash___99 | Haifahamiki
33	injuries_crash_other<br>Show the field ONLY if:<br>[injuries_crash(90)] ='1'	C7a. Ni aina gani nyingine ya majeraha ambayo uliumia/uliyopata?	text
34	hosp_crash<br>Show the field ONLY if:<br>[injured_crash]='1'	C8. Je ulilazwa hospitalini kwa ajili ya kuumia kwako?	radio<br>0 | Hapana<br>1 | Ndiyo<br>99 | Haifahamiki<br>Custom alignment: RH
35	day_hosp_crash<br>Show the field ONLY if:<br>[hosp_crash]='1'	C8a. Je ulilazwa hospitalini kwa muda wa siku ngapi?<br>*If they report being hospitalized for more then 1 year type '365'*	text (number, Min: 1, Max: 365)
36	disability_crash<br>Show the field ONLY if:<br>[injured_crash]='1'	C9. Je,ulipata ulemavu au madhara ya kudumu kutokana na ajali hiyo ?<br>*Disability would be any injury which caused chronic pain an injury requiring a change of jobs or altering daily activities*	radio<br>0 | Hapana<br>1 | Ndiyo<br>99 | Haifahamiki<br>Custom alignment: RH

37	type_disability_crash<br>Show the field ONLY if:<br>[disability_crash]='1'	C9a. Ni aina gani ya ulemavu ambao uliupata?	dropdown<br>0 Maumivu sugu ya kila siku<br>1 Majeraha yanayosababisha kubadili kazi<br>2 Mjeraha yanayosababisha kubadilisha maisha ya kila siku<br>90 Nyingine(Fafanua)
38	type_disability_other<br>Show the field ONLY if:<br>[type_disability_crash] ='90'	C9b. Ni aina gani nyingine ya ulemavu ambao uliupata?	text
39	missed_work_crash<br>Show the field ONLY if:<br>[injured_crash]='1'	C10. Je, ni siku ngapi ulikuwa nje ya kazi kutokana na majeraha hayo?<br>*If they report being out of work for more than 1 year type '365'*	text (number, Min: 1, Max: 365)
40	rehabrec_crash<br>Show the field ONLY if:<br>[injured_crash]='1'	C11. Je, rehabilitation ilipendekezwa ifanyike kwako?<br>*Rehabilitation is where a healthcare professional works with you in order to improve your functioning after an injury.*	radio<br>0 Hapana<br>1 Ndiyo<br>99 Haifahamiki<br>Custom alignment: RH
41	rehab_crash<br>Show the field ONLY if:<br>[injured_crash]='1'	C12. Je, ulipelekwa/pata rehabilitation kwa ajili ya majeraha yako?<br>*Rehabilitation is where a healthcare professional works with you in order to improve your functioning after an injury.*	radio<br>0 Hapana<br>1 Ndiyo<br>99 Haifahamiki<br>Custom alignment: RH
42	crash_year<br>Show the field ONLY if:<br>[crash_lifetime]='1'	C13. Katika mwaka uliopita uliwahi kuhusika katika ajali ya barabarani	dropdown<br>0 Hapana<br>1 Ndiyo<br>99 Haifahamiki<br>Custom alignment: RH
43	number_crash_yr<br>Show the field ONLY if:<br>[crash_year]='1'	C14. Katika mwaka uliopita ulihusika katika ajali ngapi?	text (number, Min: 1, Max: 50)
44	rem_date_ycrash<br>Show the field ONLY if:<br>[crash_year]='1'	C15. Je, unakumbuka tarehe ya ajali mbaya zaidi ?	yesno<br>1 Yes<br>0 No<br>Custom alignment: RH

45	date_ycrash<br>Show the field ONLY if:<br>[rem_date_ycrash] ="1"	C15a .Ajali yako ya hivi karibuni ilikuwa ni tarehe ngapi?<br>*ddmmyyyy*	text (date_dmy, Min: 1960-01-01, Max: 2014-02-01), Identifier
46	day_ycrash<br>Show the field ONLY if:<br>[rem_date_ycrash] ="0"	C15b. Ajali yako ya hivi karibuni ilikuwa ni siku gani?	dropdown<br>0 Jumatatu<br>1 Jumanne<br>2 Jumatano<br>3 Alhamisi<br>4 Ijumaa<br>5 Jumamosi<br>6 Jumapili<br>7 Haifahamiki
47	time_ycrash<br>Show the field ONLY if:<br>[crash_year]='1'	C16 . Ajali ilitokea mda gani?	dropdown<br>0 Asubuhi<br>1 Mchana<br>2 Jioni<br>3 Usiku<br>99 Haifahamiki
48	type_ycrash<br>Show the field ONLY if:<br>[crash_year]='1'	C17 . Ni kwa namna gani ulihusika katika ajali hiyo?	dropdown<br>0 Mtembea kwa miguu<br>1 Mpanda baisikeli<br>2 Dereva wa pikipiki ya magurudumu-3(bajaji)<br>3 Abiria wa pikipiki ya magurudumu-3(Bajaji)<br>4 Dereva wa bodaboda<br>5 Abiria wa bodaboda<br>6 Dereva wa gari dogo<br>7 Abiria wa gari dogo<br>8 dereva wa basi<br>9 Abiria wa basi<br>10 dereva wa lori<br>11 Abiria wa lori<br>12 Dala-Dala Driver<br>13 Dala-Dala Passenger<br>90 Nyingine(fafanua)<br>99 Hifahamiki

49	type_ycrash_other<br>Show the field ONLY if:<br>[time_ycrash]="90"	C17a . Ni aina ipi nyingine ya ajali ambayo umewahi kuhusika?	text
50	wk_ycrash<br>Show the field ONLY if:<br>[crash_year]='1'	C18 . Je,Ulikuwa kazini wakati ulipopata ajali hiyo?	radio<br>0 Hapana<br>1 Ndiyo<br>99 Haifahamiki<br>Custom alignment: RH
51	injured_ycrash<br>Show the field ONLY if:<br>[crash_year]='1'	C19 . Je, katika ajali hiyo uliumia/ulipata majeraha?	radio<br>0 Hapana<br>1 Ndiyo<br>99 Haifahamiki<br>Custom alignment: RH
52	hosp_ycrash<br>Show the field ONLY if:<br>[injured_ycrash]='1'	C20 . Je ulilazwa hospitalini kwa sababu ya kuumia kwako?	radio<br>0 Hapana<br>1 Ndiyo<br>99 Haifahamiki<br>Custom alignment: RH
53	day_hosp_ycrash<br>Show the field ONLY if:<br>[hosp_ycrash] = '1'	C20a . Je ulilazwa hospitalini kwa muda wa siku ngapi?<br>*If they report being hospitalized for more then 1 year type '365'*	text (number, Min: 1, Max: 365)

54	injuries_ycrash<br>Show the field ONLY if:<br>[injured_ycrash]='1'	C21 . Ni aina gani ya majeraha ambayo uliumia/uliyopata?	checkbox<br>0 | injuries_ycrash___0 | Majeraha ya kichwani<br>1 | injuries_ycrash___1 | Majeraha ya usoni<br>2 | injuries_ycrash___2 | Majeraha ya uti wa mgongo<br>3 | injuries_ycrash___3 | Majeraha ya juu juu ya kifuani<br>4 | injuries_ycrash___4 | Majeraha ya kuchimbika kwenye kifua<br>5 | injuries_ycrash___5 | Vidonda vya juu juu katika fumbatio/tumb<br>6 | injuries_ycrash___6 | Vidonda vya kuchimbika katika fumbatio/tumb<br>7 | injuries_ycrash___7 | Kuvunjika mikono<br>8 | injuries_ycrash___8 | Kuvunjika migu<br>9 | injuries_ycrash___9 | Kukatika mikon<br>10 | injuries_ycrash___10 | Kukatika miguu<br>90 | injuries_ycrash___90 | Nyingine(fafanu<br>99 | injuries_ycrash___99 | Haifahamiki
55	injuries_ycrash_other<br>Show the field ONLY if:<br>[injuries_ycrash(90)] = "1"	C21a . Ni aina gani nyingine ya majeraha ambayo uliumia?	text
56	disability_ycrash<br>Show the field ONLY if:<br>[injured_ycrash]='1'	C22 . Je,ulipata ulemavu au madhara ya kudumu kutokana na ajali hiyo?<br>*Disability would be any injury which caused chronic pain an injury requiring a change of jobs or altering daily activities*	radio<br>0 | Hapana<br>1 | Ndiyo<br>99 | Haifahamiki<br>Custom alignment: RH
57	missed_work_ycrash<br>Show the field ONLY if:<br>[injured_ycrash]='1'	C23 . Je, ni siku ngapi ulikuwa nje ya kazi kutokana na majeraha hayo?<br>*If they report being out of work for more than 1 year type '365'*	text (number, Min: 1, Max: 365)
58	rehabrec_ycrash<br>Show the field ONLY if:<br>[injured_ycrash]='1'	C24 . Je, ilipendekezwa ufanyiwe rehabilitation?<br>*Rehabilitation is where a healthcare professional works with you in order to improve your functioning after an injury.*	radio<br>0 | Hapana<br>1 | Ndiyo<br>99 | Haifahamiki<br>Custom alignment: RH

59	rehab_ycrash<br><br>Show the field ONLY if:<br>[injured_ycrash]='1'	C25 . Je, ulifanyiwa/pata rehabilitation kwa ajili ya majeraha yako?<br>*Rehabilitation is where a healthcare professional works with you in order to improve your functioning after an injury.*	radio<br>0 Hapana<br>1 Ndiyo<br>99 Haifahamiki<br><br>Custom alignment: RH
60	near_miss_month<br><br>Show the field ONLY if:<br>[consent]='1'	C26 . Je, ni mara ngapi umekaribia kupata ajali lakini hukupata katika mwezi uliopita?<br>*A near-miss crash is one in which you thought you were going to crash but did not*	radio<br>0 0<br>1 1-5<br>2 5-10<br>3 10-15<br>4 >15<br><br>Custom alignment: RH
61	crash_opinion_other<br><br>Show the field ONLY if:<br>[consent]='1'	C27 . Kwa maoni yako,unafikiri nini kingefanyika ili kupunguza ajali za bodaboda?<br>*Do not give them ideas, try to see what they will answer without assistance. If they say "I don't know" you can simply type 'IDK'*	text

62	crash_opinion<br><br>Show the field ONLY if:<br>[consent]='1'	C27a . Je, unafikiri kwamba kuna mapendekezo yoyote kati ya haya yafuatayo yangeweza kupunguza idadi ya ajali za bodaboda?	checkbox<br>0 | crash_opinion___0 | Kutengeneza barabara kwa ajaili ya bodaboda<br>1 | crash_opinion___1 | Kuimarisha hali ya barabara<br>2 | crash_opinion___2 | Kupunguza msongamano barabarani<br>3 | crash_opinion___3 | kuongeza taratibu/kanunu za barabarani<br>4 | crash_opinion___4 | kufanya vyomo vingine vyo barabarani kuwa makini na watu wa bodaboda<br>5 | crash_opinion___5 | Kuongeza njia za watembea kwa miguu<br>6 | crash_opinion___6 | Kuboresha mafundisho kwa madereva wa bodaboda<br>7 | crash_opinion___7 | Kuwe na taa Nzuri za barabarani<br>8 | crash_opinion___8 | Kuwe na fulana akisi kwa Madereva<br>9 | crash_opinion___9 | Kuwe na ulazima wa kuvaa helment<br>10 | crash_opinion___10 | Nyingine (Fafanua)<br>11 | crash_opinion___11 | Hakuna.
63	crash_other<br><br>Show the field ONLY if:<br>[consent]='1'	C27b. Je,unafikiri ni aina zipi nyingine zingeweza kutatua/kupunguza ajali za badaboda?	text
64	crash_boda_boda<br><br>Show the field ONLY if:<br>[consent]='1'	C28 . Je, unafikiri kwamba bodaboda ndio kisababishi cha ajali nyingi za barabarani katika mji wa Moshi?	dropdown<br>98 | N/A<br>0 | Hapana<br>1 | Mara chache<br>2 | Wakati fulani<br>3 | Mara kwa mara<br>4 | Mara zote

65	crash_car<br><br>Show the field ONLY if:<br>[consent]='1'	C29 . Je, unafikiri kwamba magari madogo ndio kisababishi cha ajali nyingi za barabarani katika mji wa Moshi?	dropdown<br>98 N/A<br>0 Hapana<br>1 Mara chache<br>2 Wakati fulani<br>3 Mara kwa mara<br>4 Mara zote
66	crash_bus<br><br>Show the field ONLY if:<br>[consent]='1'	C30 . Je, unafikiri kwamba mabasi ndio kisababishi cha ajali nyingi za barabarani katika mji wa Moshi?	dropdown<br>98 N/A<br>0 Hapana<br>1 Mara chache<br>2 Wakati fulani<br>3 Mara kwa mara<br>4 Mara zote
67	crash_peds<br><br>Show the field ONLY if:<br>[consent]='1'	C31 . Je, unafikiri kwamba watembea kwa miguu ndio kisababishi cha ajali nyingi za barabarani katika mji wa Moshi?	dropdown<br>98 N/A<br>0 Hapana<br>1 Mara chache<br>2 Wakati fulani<br>3 Mara kwa mara<br>4 Mara zote
68	crash_bus2<br><br>Show the field ONLY if:<br>[consent]='1'	C32. Je, unafikiri kwamba daladala ndio kisababishi cha ajali nyingi za barabarani katika mji wa Moshi?	dropdown<br>98 N/A<br>0 Hapana<br>1 Mara chache<br>2 Wakati fulani<br>3 Mara kwa mara<br>4 Mara zote
69	helmet_mc<br><br>Show the field ONLY if:<br>[consent]='1'	Section Header: *Personal Protective Equipment*<br><br>P1. Ni mara ngapi huwa unavaa kofia ngumu wakati unaendesha bodaboda?	dropdown<br>98 N/A<br>0 Hapana<br>1 Mara chache<br>2 Wakati fulani<br>3 Mara kwa mara<br>4 Mara zote

70	helmet_colleagues<br>Show the field ONLY if:<br>[consent]='1'	P2. Ni mara ngapi madereva wenzako wa bodaboda huwa wanavaa kofia ngumu wakati wanaendesha bodaboda?	dropdown<br>98 N/A<br>0 Hapana<br>1 Mara chache<br>2 Wakati fulani<br>3 Mara kwa mara<br>4 Mara zote
71	colleagues_risks<br>Show the field ONLY if:<br>[consent]='1'	P3 . Ni mara ngapi madereva wenzako wa bodaboda wanajihatarisha wakati wanapoendesha bodaboda?	dropdown<br>98 N/A<br>0 Hapana<br>1 Mara chache<br>2 Wakati fulani<br>3 Mara kwa mara<br>4 Mara zote
72	headlights_always<br>Show the field ONLY if:<br>[consent]='1'	P6 . Je huwa unawasha taa kubwa wakati wote unapoendesha bodaboda yako?	dropdown<br>98 N/A<br>0 Hapana<br>1 Mara chache<br>2 Wakati fulani<br>3 Mara kwa mara<br>4 Mara zote
73	headlights_night<br>Show the field ONLY if:<br>[consent]='1'	P7 . Je huwa unawasha taa kubwa unapoendesha bodaboda yako gizani(usiku)?	dropdown<br>98 N/A<br>0 Hapana<br>1 Mara chache<br>2 Wakati fulani<br>3 Mara kwa mara<br>4 Mara zote
74	helmet_damage<br>Show the field ONLY if:<br>[consent]='1'	P8 . Je, madereva wa bodaboda hununua kofia ngumu(Helmeti) mpya baada ya ajali au kama imeharibika?	dropdown<br>98 N/A<br>0 Hapana<br>1 Mara chache<br>2 Wakati fulani<br>3 Mara kwa mara<br>4 Mara zote

75	helmet_strap_use<br>Show the field ONLY if:<br>[consent]='1'	P9 . Je, wewe huwa unafunga mkanda wa kofia ngumu (helmeti) wakati unapoivaa/unapoitumia?	dropdown<br>98 N/A<br>0 Hapana<br>1 Mara chache<br>2 Wakati fulani<br>3 Mara kwa mara<br>4 Mara zote
76	helmet_value<br>Show the field ONLY if:<br>[consent]='1'	P10 . Je, unaamini kwamba kofia ngumu (Helmet) inapunguza majeraha wakati wa ajali?	dropdown<br>98 N/A<br>1 Sikubali kabisa<br>2 Sikubali<br>3 Siko upande wowote<br>4 Nakubali<br>5 Nakubali kabisa
77	helmet_strap_value<br>Show the field ONLY if:<br>[consent]='1'	P11 . Je, unaamini kwamba mkanda katika kofia ngumu(Helmet) ni muhimu katika kupunguza majeraha katika ajali?<br>*Please report the number in Tanzanian Shillings*	dropdown<br>98 N/A<br>1 Sikubali kabisa<br>2 Sikubali<br>3 Siko upande wowote<br>4 Nakubali<br>5 Nakubali kabisa
78	helmet_expensive<br>Show the field ONLY if:<br>[consent]='1'	P12 .Je Unafikiri kofia ngumu (Helmeti) mpya inatakiwa iuzwe shilingi ngapi?	text (number, Min: 200, Max: 1000000)
79	helmet_cost_used<br>Show the field ONLY if:<br>[consent]='1'	P13 . Je kofia ngumu iliyotumika inagharimu/inauzwa shilingi ngapi?	text (number, Min: 200, Max: 1000000)
80	helmet_cost_new<br>Show the field ONLY if:<br>[consent]='1'	P14 . Je kofia ngumu mpya inagharimu/inauzwa shilingi ngapi?	text (number, Min: 200, Max: 1000000)
81	helmet_where_buy<br>Show the field ONLY if:<br>[consent]='1'	P15 .Je duka unalonunua kofia ngumu(Helmeti) hapa Moshi linaitwaje na liko sehemu gani ya mji wa Moshi?	text
82	crash_helmet<br>Show the field ONLY if:<br>[consent]='1'	P16 . Je umewahi kupata ajali wakati ukiwa umevaa kofia yako ngumu yoyote?	yesno<br>1 Yes<br>0 No<br>Custom alignment: RH

83	desc<br>Show the field ONLY if:<br>[consent]='1'	Tafadhali ninaweza kuiangalia kofia yako ngumu ambayo huwa unaivaa wakati unapoendesha bodaboda?. (Ikamate kofia ngumu kwenye mikono yako na kisha ikague kwa makini kwa kuangalia nyufa juu ya ganda,kama imeharibika sehemu yoyote,mikwaruzo,uchafu ulioganda,rangi juu ya kinga ya uso au matatizo kwenye mkanda wa kidevuni/kifungio.Baada ya kuikagua kofia ngumu wewe mwenyewe jibu maswali yote yanayofuata chini kwa kuzingatia kile ulichogundua, (usimuulize dereva wa bodaboda).<br>*Look for signs of serious damage to the helmet, it should normally have no cracks or dents. If you find some cracks or dents or other serious damage you should choose 'Yes'.*	descriptive
84	cracks_dhelmet<br>Show the field ONLY if:<br>[consent]='1'	P17 . Je, kofia ngumu (Helmeti) ya dereva ina nyufa au mbonyeo kwenye ganda lake?<br>*Look for areas with larger scratches or areas of paint removed, if it appears that the helmet has been damaged choose 'Yes'.*	yesno<br>1 Yes<br>0 No<br>Custom alignment: RH
85	scratches_dhelmet<br>Show the field ONLY if:<br>[consent]='1'	P18 . Je,kofia ngumu (Helmeti) ya dereva ina mikwaruzo katika rangi?<br>*Open and close the chin strap to see if the buckle is broken. Try to adjust the size of the strap. If you are NOT able to buckle or adjust the strap you should choose 'Yes'*	yesno<br>1 Yes<br>0 No<br>Custom alignment: RH
86	strap_dhelmet<br>Show the field ONLY if:<br>[consent]='1'	P19 . Je, kofia ngumu (helmeti) ya dereva imekatika/imevunjika mkanda wa kidevuni?	yesno<br>1 Yes<br>0 No<br>Custom alignment: RH
87	face_shield<br>Show the field ONLY if:<br>[consent]='1'	P20 . Je, kuna kinga/ngao ya usoni?<br>*Try to look through the face shield of the helmet as if you were wearing it. If it is NOT clear and easy to see through choose 'Yes'*	yesno<br>1 Yes<br>0 No<br>Custom alignment: RH
88	glass_helmet<br>Show the field ONLY if:<br>[consent]='1'	P20a. Je, ngao katika kofia ngumu(Helmeti) ya dereva imefifishwa na mikwaruzo, rangi au michoro?<br>*Ask the driver to put the helmet on and then shake their head around a bit so you can judge if the helmet seems to fit well or if it moves around a lot of the drivers head. If the helmet does not move a lot choose 'Yes'*	yesno<br>1 Yes<br>0 No<br>Custom alignment: RH
89	fit_helmet<br>Show the field ONLY if:<br>[consent]='1'	P21 . Je, kofia ngumu (Helmeti) ya dereva inamuenea vizuri?<br>*Try to get the boda-boda to report 10 locations. If they say something like "crashes happen everywhere" you should push them to tell you some specific spots where they have seen crashes happen. If they are having trouble you may ask them to tell you about a spot where they have seen even one crash occur.*	yesno<br>1 Yes<br>0 No<br>Custom alignment: RH

90	desc2<br>Show the field ONLY if:<br>[consent]='1'	Section Header: *High Risk Road Intersections*<br>Tafadhali naomba unitajie mahali ambapo umeona ajali nyingi zinatokea,au eneo la jirani.Elezea mahali hapo kwa mtaa au kijiji na pia taja jina la barabara kama inawezekana na alama ya kudumu ambayo inatambulisha mahali hapo.Tafadhali ipe kiwango cha hatari kwa kila mahali kwa kipimo cha 0 (Hakuna hatari) mpaka 100 (Hatari zaidi).<br>*Get as much detail as possible like cell or village or common name of location*	descriptive
91	site1<br>Show the field ONLY if:<br>[consent]='1'	DL1. Sehemu husika na mazingira ya jirani:<br>*Ask about the type of road the location is on (bend, junction, roundabout, etc) and any nearby buildings/places that could help to identify the spot*	text, Identifier
92	refpt_1<br>Show the field ONLY if:<br>[consent]='1'	DL1. Alama ya kumbukumbu:	text, Identifier
93	lat_1<br>Show the field ONLY if:<br>[consent]='1'	DL1 . Latitudo ya alama	text (number, Min: -3.39, Max: -3.3), Identifier
94	long_1<br>Show the field ONLY if:<br>[consent]='1'	DL1 . Longitudo ya alama<br>*0 - No danger | 100 - Most dangerous*	text (number, Min: 37.29, Max: 37.41), Identifier
95	danger1<br>Show the field ONLY if:<br>[consent]='1'	DL1 . Ni hatari kwa kiasi gani katika kipimo cha 0 (hakuna hatari) kwa 100 (hatari zaidi) Katika eneo hili?<br>*Get as much detail as possible like cell or village or common name of location*	text (integer, Min: 0, Max: 100)
96	site2<br>Show the field ONLY if:<br>[site1] <> ''	DL2. Sehemu husika na mazingira ya jirani:<br>*Ask about the type of road the location is on (bend, junction, roundabout, etc) and any nearby buildings/places that could help to identify the spot*	text, Identifier
97	refpt_2<br>Show the field ONLY if:<br>[site1] <> ''	DL2. Alama ya kumbukumbu:	text, Identifier
98	lat_2<br>Show the field ONLY if:<br>[site1] <> ''	DL2. Latitudo ya alama	text (number, Min: -3.39, Max: -3.3), Identifier
99	long_2<br>Show the field ONLY if:<br>[site1] <> ''	DL2. Longitudo ya alama<br>*0 - No danger | 100 - Most dangerous*	text (number, Min: 37.29, Max: 37.41), Identifier

100	danger2<br>Show the field ONLY if:<br>[site1] <> ''	DL2 . Ni hatari kwa kiasi gani katika kipimo cha 0 (hakuna hatari) kwa 100 (hatari zaidi) Katika eneo hili?<br>*Get as much detail as possible like cell or village or common name of location*	text (integer, Min: 0, Max: 100)
101	site3<br>Show the field ONLY if:<br>[site2] <> ''	DL3. Sehemu husika na mazingira ya jirani:<br>*Ask about the type of road the location is on (bend, junction, roundabout, etc) and any nearby buildings/places that could help to identify the spot*	text, Identifier
102	refpt_3<br>Show the field ONLY if:<br>[site2] <> ''	DL3. Alama ya kumbukumbu:	text, Identifier
103	lat_3<br>Show the field ONLY if:<br>[site2] <> ''	DL3. Latitudo ya alama	text (number, Min: -3.39, Max: -3.3), Identifier
104	long_3<br>Show the field ONLY if:<br>[site2] <> ''	DL3. Longitudo ya alama<br>*0 - No danger | 100 - Most dangerous*	text (number, Min: 37.29, Max: 37.41), Identifier
105	danger3<br>Show the field ONLY if:<br>[site2] <> ''	DL3 . Ni hatari kwa kiasi gani katika kipimo cha 0 (hakuna hatari) kwa 100 (hatari zaidi) Katika eneo hili?<br>*Get as much detail as possible like cell or village or common name of location*	text (integer, Min: 0, Max: 100)
106	site4<br>Show the field ONLY if:<br>[site3] <> ''	DL4. Sehemu husika na mazingira ya jirani:<br>*Ask about the type of road the location is on (bend, junction, roundabout, etc) and any nearby buildings/places that could help to identify the spot*	text, Identifier
107	refpt_4<br>Show the field ONLY if:<br>[site3] <> ''	DL4. Alama ya kumbukumbu:	text, Identifier
108	lat_4<br>Show the field ONLY if:<br>[site3] <> ''	DL4. Latitudo ya alama	text (number, Min: -3.39, Max: -3.3), Identifier
109	long_4<br>Show the field ONLY if:<br>[site3] <> ''	DL4. Longitudo ya alama<br>*0 - No danger | 100 - Most dangerous*	text (number, Min: 37.29, Max: 37.41), Identifier
110	danger4<br>Show the field ONLY if:<br>[site3] <> ''	DL4 . Ni hatari kwa kiasi gani katika kipimo cha 0 (hakuna hatari) kwa 100 (hatari zaidi) Katika eneo hili?<br>*Get as much detail as possible like cell or village or common name of location*	text (integer, Min: 0, Max: 100)
111	site5<br>Show the field ONLY if:<br>[site4] <> ''	DL5. Sehemu husika na mazingira ya jirani:<br>*Ask about the type of road the location is on (bend, junction, roundabout, etc) and any nearby buildings/places that could help to identify the spot*	text, Identifier

112	refpt_5<br><br>Show the field ONLY if:<br>[site4] <> ''	DL5. Alama ya kumbukumbu:	text, Identifier
113	lat_5<br><br>Show the field ONLY if:<br>[site4] <> ''	DL5. Latitudo ya alama	text (number, Min: -3.39, Max: -3.3), Identifier
114	long_5<br><br>Show the field ONLY if:<br>[site4] <> ''	DL5. Longitudo ya alama<br>*0 - No danger | 100 - Most dangerous*	text (number, Min: 37.29, Max: 37.41), Identifier
115	danger5<br><br>Show the field ONLY if:<br>[site4] <> ''	DL5 . Ni hatari kwa kiasi gani katika kipimo cha 0 (hakuna hatari) kwa 100 (hatari zaidi) Katika eneo hili?<br>*Get as much detail as possible like cell or village or common name of location*	text (integer, Min: 0, Max: 100)
116	site6<br><br>Show the field ONLY if:<br>[site5] <> ''	DL6. Sehemu husika na mazingira ya jirani:<br>*Ask about the type of road the location is on (bend, junction, roundabout, etc) and any nearby buildings/places that could help to identify the spot*	text, Identifier
117	refpt_6<br><br>Show the field ONLY if:<br>[site5] <> ''	DL6. Alama ya kumbukumbu:	text, Identifier
118	lat_6<br><br>Show the field ONLY if:<br>[site5] <> ''	DL6. Latitudo ya alama	text (number, Min: -3.39, Max: -3.3), Identifier
119	long_6<br><br>Show the field ONLY if:<br>[site5] <> ''	DL6. Longitudo ya alama<br>*0 - No danger | 100 - Most dangerous*	text (number, Min: 37.29, Max: 37.41), Identifier
120	danger6<br><br>Show the field ONLY if:<br>[site5] <> ''	DL6 . Ni hatari kwa kiasi gani katika kipimo cha 0 (hakuna hatari) kwa 100 (hatari zaidi) Katika eneo hili?<br>*Get as much detail as possible like cell or village or common name of location*	text (integer, Min: 0, Max: 100)
121	site7<br><br>Show the field ONLY if:<br>[site6] <> ''	DL7. Sehemu husika na mazingira ya jirani:<br>*Ask about the type of road the location is on (bend, junction, roundabout, etc) and any nearby buildings/places that could help to identify the spot*	text, Identifier
122	refpt_7<br><br>Show the field ONLY if:<br>[site6] <> ''	DL7. Alama ya kumbukumbu:	text, Identifier
123	lat_7<br><br>Show the field ONLY if:<br>[site6] <> ''	DL7. Latitudo ya alama	text (number, Min: -3.39, Max: -3.3), Identifier

124	long_7<br><br>Show the field ONLY if:<br>[site6] <> ''	DL7. Longitudo ya alama<br>*0 - No danger | 100 - Most dangerous*	text (number, Min: 37.29, Max: 37.41), Identifier
125	danger7<br><br>Show the field ONLY if:<br>[site6] <> ''	DL7 . Ni hatari kwa kiasi gani katika kipimo cha 0 (hakuna hatari) kwa 100 (hatari zaidi) Katika eneo hili?<br>*Get as much detail as possible like cell or village or common name of location*	text (integer, Min: 0, Max: 100)
126	site8<br><br>Show the field ONLY if:<br>[site7] <> ''	DL8. Sehemu husika na mazingira ya jirani:<br>*Ask about the type of road the location is on (bend, junction, roundabout, etc) and any nearby buildings/places that could help to identify the spot*	text, Identifier
127	refpt_8<br><br>Show the field ONLY if:<br>[site7] <> ''	DL8. Alama ya kumbukumbu:	text, Identifier
128	lat_8<br><br>Show the field ONLY if:<br>[site7] <> ''	DL8. Latitudo ya alama	text (number, Min: -3.39, Max: -3.3), Identifier
129	long_8<br><br>Show the field ONLY if:<br>[site7] <> ''	DL8. Longitudo ya alama<br>*0 - No danger | 100 - Most dangerous*	text (number, Min: 37.29, Max: 37.41), Identifier
130	danger8<br><br>Show the field ONLY if:<br>[site7] <> ''	DL8 . Ni hatari kwa kiasi gani katika kipimo cha 0 (hakuna hatari) kwa 100 (hatari zaidi) Katika eneo hili?<br>*Get as much detail as possible like cell or village or common name of location*	text (integer, Min: 0, Max: 100)
131	site9<br><br>Show the field ONLY if:<br>[site8] <> ''	DL9. Sehemu husika na mazingira ya jirani:<br>*Ask about the type of road the location is on (bend, junction, roundabout, etc) and any nearby buildings/places that could help to identify the spot*	text, Identifier
132	refpt_9<br><br>Show the field ONLY if:<br>[site8] <> ''	DL9. Alama ya kumbukumbu:	text, Identifier
133	lat_9<br><br>Show the field ONLY if:<br>[site8] <> ''	DL9. Latitudo ya alama	text (number, Min: -3.39, Max: -3.3), Identifier
134	long_9<br><br>Show the field ONLY if:<br>[site8] <> ''	DL9. Longitudo ya alama<br>*0 - No danger | 100 - Most dangerous*	text (number, Min: 37.29, Max: 37.41), Identifier
135	danger9<br><br>Show the field ONLY if:<br>[site8] <> ''	DL9 . Ni hatari kwa kiasi gani katika kipimo cha 0 (hakuna hatari) kwa 100 (hatari zaidi) Katika eneo hili?<br>*Get as much detail as possible like cell or village or common name of location*	text (integer, Min: 0, Max: 100)

136	site10<br>Show the field ONLY if:<br>[site9] <> ''	DL10. Sehemu husika na mazingira ya jirani:<br>*Ask about the type of road the location is on (bend, junction, roundabout, etc) and any nearby buildings/places that could help to identify the spot*	text, Identifier
137	refpt_10<br>Show the field ONLY if:<br>[site9] <> ''	DL10. Alama ya kumbukumbu:	text, Identifier
138	lat_10<br>Show the field ONLY if:<br>[site9] <> ''	DL10. Latitudo ya alama	text (number, Min: -3.39, Max: -3.3), Identifier
139	long_10<br>Show the field ONLY if:<br>[site9] <> ''	DL10. Longitudo ya alama<br>*0 - No danger | 100 - Most dangerous*	text (number, Min: 37.29, Max: 37.41), Identifier
140	danger10<br>Show the field ONLY if:<br>[site9] <> ''	DL10 . Ni hatari kwa kiasi gani katika kipimo cha 0 (hakuna hatari) kwa 100 (hatari zaidi) Katika eneo hili?	text (integer, Min: 0, Max: 100)
141	thanks2<br>Show the field ONLY if:<br>[consent]='1'	Ahsante kwa kushiriki katika utafiri huu. Matokeo ya utafiti huu yatapatikana katika kitengo cha utafiti cha hospitali ya KCMC. Tafadhali jisikie huru kuniuliza swali lolote kuhusu utafiti huu. (kama hakuna maswali ... Ahsante kwa ushirikiano wako, Nakutakia siku njema!)	descriptive
142	pilot_complete	Section Header: *Form Status*<br>Complete?	dropdown<br>0 Incomplete<br>1 Unverified<br>2 Complete
